# Localized *rbp4 *expression in the yolk syncytial layer plays a role in yolk cell extension and early liver development

**DOI:** 10.1186/1471-213X-7-117

**Published:** 2007-10-19

**Authors:** Zhen Li, Vladimir Korzh, Zhiyuan Gong

**Affiliations:** 1 Department of Biological Sciences, National University of Singapore, Singapore; 2 Laboratory of Fish Developmental Biology, Institute of Molecular and Cell Biology, Singapore; 3 Computation and System Biology Program, Singapore-MIT Alliance, National University of Singapore, Singapore

## Abstract

**Background:**

The number of genes characterized in liver development is steadily increasing, but the origin of liver precursor cells and the molecular control of liver formation remain poorly understood. Existing theories about formation of zebrafish visceral organs emphasize either their budding from the endodermal rod or formation of independent anlage followed by their later fusion, but none of these is completely satisfactory in explaining liver organogenesis in zebrafish.

**Results:**

Expression of a gene encoding the retinol binding protein 4 (Rbp4) was analyzed in zebrafish. *rbp4*, which is expressed mainly in the liver in adults, was shown to be expressed in the yolk syncytial layer (YSL) during early embryogenesis. At 12–16 hpf *rbp4 *expression was restricted to the ventro-lateral YSL and later expanded to cover the posterior YSL. We demonstrated that *rbp4 *expression was negatively regulated by Nodal and Hedgehog (Hh) signalling and positively controlled by retinoic acid (RA). Knockdown of Rbp4 in the YSL resulted in shortened yolk extension as well as the formation of two liver buds, which could be due to impaired migration of liver progenitor cells. *rbp4 *appears also to regulate the extracellular matrix protein Fibronectin1 (Fn1) specifically in the ventro-lateral yolk, indicating a role of Fn1 in liver progenitor migration. Since exocrine pancreas, endocrine pancreas, intestine and heart developed normally in Rbp4 morphants, we suggest that *rbp4 *expression in the YSL is required only for liver development.

**Conclusion:**

The characteristic expression pattern of *rbp4 *suggests that the YSL is patterned despite its syncytial nature. YSL-expressed Rbp4 plays a role in formation of both yolk extension and liver bud, the latter may also require migration of liver progenitor cells.

## Background

The YSL is an extra-embryonic structure and it forms at the stage of mid-blastula transition (MBT) in teleosts [1,2] by a poorly understood developmental mechanism. The YSL performs several early developmental functions such as yolk metabolism, nutrient transport [3], utilization of maternally stored morphogenetic substances including retinoids [4,5], and epiboly movement [6]. It also plays a morphogenetic role during gastrulation in induction and patterning of mesoderm, endoderm and dorsal structures [7-13]. However, there is little information on the function of the YSL after epiboly is completed. Recently it has been reported that the YSL-specific factor Mtx1 plays a role in migration of myocardial precursor cells and knockdown of Mtx1 in the YSL resulted in *cardia bifida *and duplication of liver and pancreatic buds. Based on these results it has been proposed that the YSL regulates migration of endodermal cells [14].

Rbp4 is produced in the adult liver and functions as a specific transporter of retinol in vertebrate plasma. Expression of *rbp4 *has been studied in several model animals. During embryonic development of rodents it is expressed only in the visceral extra-embryonic endoderm of the yolk sac, suggesting that Rbp4 may play roles in mediating retinol transfer from maternal blood to the developing fetus [15,16]. Similar expression has also been reported in chick [17]. In zebrafish, *rbp4 *has been reported to be expressed in the YSL, hypochord and skin [18]. It is of interest to analyse the role of *rbp4 *in zebrafish development.

The liver is an important endodermal organ, which exerts both endocrine and exocrine functions. Many studies have revealed that the molecular mechanisms of liver development are conserved in vertebrates [19-21]. Furthermore, cell fate mapping experiments in zebrafish, frog and mouse have also indicated that the liver arises, at least in part, from different groups of endodermal cells found initially in bilateral regions on both sides of the midline [22-24]. Despite this progress the mechanism of liver bud formation in zebrafish is not fully understood. According to one hypothesis based to a large extent on observations of gutGFP transgenic zebrafish, the early endoderm forms as an endodermal rod, which starts to bud and gives rise to several primordia including the liver primordium which grows mainly due to cell proliferation within the primordium [25,26]. A conflicting view based on analysis of different molecular markers implies that whereas the digestive anlagen of amniotes arise from a primitive gut tube, the zebrafish digestive system is assembled from individual organ anlage [27]. In addition, based on analysis of the expression pattern of *ceruloplasmin *in wild type and mutant zebrafish, migration of progenitor cells from posterior to anterior and towards the midline has been proposed to take place during formation of the liver [28]. Recently, this hypothesis has been supported by discovery of the posterior-to-anterior migration of cells between the enveloping layer (EVL) and YSL, which is linked to the formation of the yolk cell extension (YCE; [29]).

In this study, we demonstrated that early expression of *rbp4 *in the YSL is restricted only to the ventro-lateral YSL. *rbp4 *expression is negatively controlled by Nodal and Hh signalling pathways and positively regulated by RA. The YSL-specific knockdown of Rbp4 caused inhibition of the YCE formation and formation of two liver buds. Thus, Rbp4 probably plays a role in morphogenesis of the yolk cell and formation of the liver bud.

## Results

### Expression of *rbp4 *mRNAs

**Figure 1:**
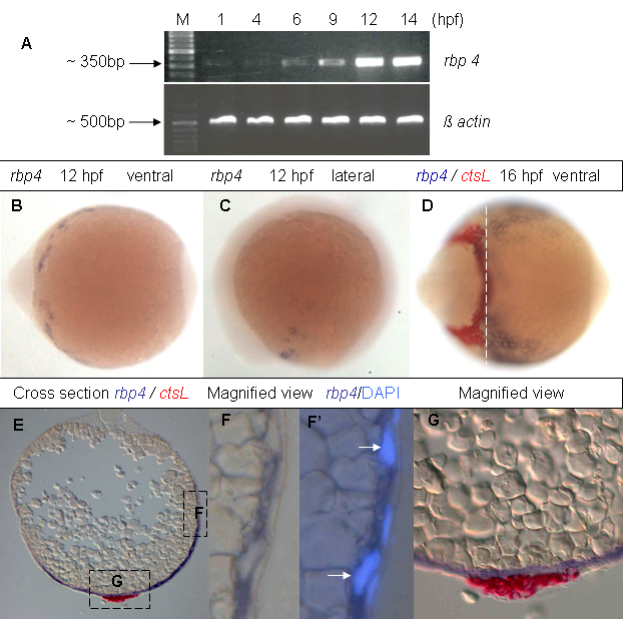
**Expression of *rbp4 *mRNA during early zebrafish embryogenesis**. (A) RT-PCR analysis of *rbp4 *mRNA in wild-type embryos from 1 hpf to 14 hpf. *β-actin *was used as loading control. M, 100 bp DNA marker. (B, C) Ventral (B) and lateral (C) view of 12 hpf embryos with *rbp4 *expression as detected by WISH. (D) Ventral view of 16 hpf embryos with expression of *ctsL *(red) and *rbp4 *(blue) as detected by two-colour WISH. (E) Cross section of the two color hybridized embyos in (D) as indicated by the dashed line. (F, F') Magnified view of boxed region F in Panel (E). F, bright field. F', compound image of DIC/fluorescence reveals *rbp4 *expression and position of nuclei detected by DAPI staining. Arrows indicate YSL nuclei. (G), Magnified view of boxed region G in Panel (E).

The EST clone coding for Rbp4 (A 10) from our collection [30] was selected and sequenced completely. The complete sequence was submitted to GenBank (acc. no. EF373650) and it was almost identical (99.5%) to a sequence previously available (GenBank AJ236884, [18]). To investigate the temporal expression pattern of *rbp4 *in early development, *rbp4 *transcripts were analyzed by RT-PCR from fertilized eggs to 14 hpf. We found that *rbp4 *transcripts were of maternal origin and the zygotic expression of *rbp4 *increased from 6 hpf to reach its peak by 12 hpf (Figure 1A).

To further investigate the pattern of *rbp4 *expression in zebrafish embryos, whole mount *in situ *hybridization (WISH) was carried out. Transcripts of *rbp4 *were detected from 12 hpf on the yolk surface anterior to the head. This expression domain was confined to the ventro-lateral yolk cell (Figure 1B, C). At 16 hpf, *rbp4 *expression increased and expanded posteriorly but remained confined to the ventro-lateral yolk cell (Figure 1D). To define this expression domain in detail, two-colour WISH using *ctsL *(*cathepsinL*, [31]) and *rbp4 *probes was performed at 16 hpf. *ctsL *was expressed in the hatching gland mesoderm around the head as expected, while the anterior end of the *rbp4 *expression domain was found to spread under the head (Figure 1D). A cross section indicated that *ctsL *and *rbp4 *were expressed in different layers of tissue (Figure 1E, G). DAPI staining clearly revealed that the enlarged nuclei, characteristic of the YSL, were surrounded by *rbp4 *transcripts (Figure 1F, F'), while the superficial cellular layer or the enveloping layer was free of *rbp4 *transcripts. Thus, the expression of *rbp*4 on the surface of the yolk cell was probably confined to the YSL. To the best of our knowledge this is the first molecular marker of the ventro-lateral YSL.

**Figure 2:**
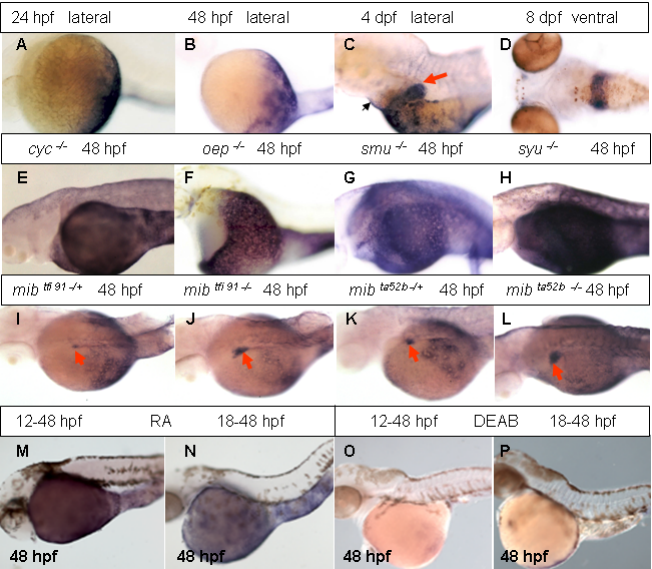
**Analyses of *rbp4 *expression during late development and its regulation**. (A-D) *rbp4 *expression in 24 hpf, 48 hpf, 4 dpf and 8 dpf wild-type embryos as indicated. A red arrow indicates the liver while a black arrow indicates the pericardium region. (E-H) *rbp4 *expression in *cyc*^-/-^, *oep*^-/-^, *smu*^-/- ^and *syu*^-/- ^embryos at 48 hpf as indicated. (I – L) *rbp4 *expression in various heterozygous and homozygous *mib *mutant embryos as indicated. Red arrows indicate the liver. Note precocious appearance of *rbp4 *expression in homozygous mutant liver at 48 hpf (J, L) compared to heterozygous *mib *embryos (I, K). (M, N) *rbp4 *expression in 48 hpf embryos treated with 10^-6 ^M RA initiated from 12 hpf (M) or 18 hpf (N). (O, P) *rbp4 *expression in 48 hpf embryos treated with 10^-5 ^M DEAB initiated from 12 hpf (O) and 18 hpf (P).

At 24 hpf, *rbp4 *expression in the YSL is restricted to its posterior part including that in the YCE (Figure 2A). This expression pattern remained unchanged at least until 48 hpf (Figure 2B). Later the expression spread anteriorly but remained excluded from the pericardium (Figure 2C). By 4 dpf, *rbp4 *expression was detected in the liver (Figure 2C). By 8 dpf the liver became the only tissue with detectable *rbp4 *expression (Figure 2D).

To understand the developmental mechanism controlling the unusual spatial expression pattern of *rbp4 *in the YSL, the expression pattern of this gene in several mutants affecting various aspects of early endoderm development was analyzed. In mutants affecting Nodal signalling (*cyc *and *oep*) (Figure 2E, F), *rbp4 *expression expanded anteriorly and covered the whole yolk. Similar changes of expression pattern were also observed in mutants that affected components of the Hh signalling pathway, *smu *(Figure 2G), *syu *(Figure 2H) and *yot *(not shown). As the restricted expression pattern of *rbp4 *was lost at 48 hpf in these mutants, it is likely that the early expression pattern of *rbp4 *in the YSL is under negative regulation of the Nodal and Hedgehog signaling pathways, which prevent the *rbp4 *expression in the anterior and dorsal YSL.

In the *mib*^*tfi91 *^mutant which has a nonsense mutation in the gene encoding E3 ubiquitin ligase involved in ubiqutination of Delta, a ligand of Notch signaling, Delta is up-regulated causing premature differentiation of neural progenitor cells [32]. The restricted *rbp4 *expression pattern in the YSL was maintained, but the expression in the liver appeared much earlier than that in control embryos (48 hpf vs. 96 hpf). In addition, the *rbp4 *expression in the liver of *mib*^*tfi91*-/+ ^heterozygotes also appeared prematurely but the expression domain was smaller than that in *mib*^*tfi91*-/- ^homozygotes (Figure 2I and 2J). Further analysis of another allele of *mib *mutant, *mib*^*ta52b*^, demonstrated that the expression domain of *rbp4 *in the liver is larger in the *mib*^*ta52b *^allele (Figure 2K and 2L) than that in the *mib*^*tfi91 *^allele. This is in accordance with the fact that *mib*^*ta52b*-/- ^allele is a stronger dominant negative mutation, while the *mib*^*tfi91*-/- ^allele is a weaker null allele [32].

Analyses of embryos of two mutant lines with defects in formation of axial mesoderm, *spt *and *ntl*, did not show obvious changes in expression pattern of *rbp4 *from 16 hpf to 24 hpf, suggesting that the formation of axial mesoderm probably does not influence events involving *rbp4 *in the YSL (not shown).

### RA positively regulates rbp4 expression in the YSL

Retinol is the precursor of RA, which plays important roles in many aspects of development [33,34]. In order to examine whether RA could regulate *rbp4 *expression, different doses of RA and DEAB (4-diethylaminobenzaldehyde), an inhibitor of RA synthesis acting through inhibition of retinaldehyde dehydrogenase [35], were applied to wild type embryos starting from 12 hpf, 16 hpf and 18 hpf, respectively. Control embryos were soaked in 0.1% DMSO/egg water. WISH on 48 hpf embryos with the *rbp4 *probe showed that in all cases RA treatment caused expansion of *rbp4 *expression (Figure 2M and 2N). In contrast, DEAB treatment caused reduction of *rbp4 *expression (Figure 2O and 2P). Thus, RA positively regulates *rbp4 *expression in the YSL. It is interesting to note that the phenotypes were more severe when the treatment was initiated early. In the RA treatment group, *rbp4 *expression was generally stronger in embryos treated from 12 hpf (Figure 2M) than that from 18 hpf (Figure 2N). In the DEAB treatment group, the yolk extension was affected more severely when the treatment was started from 12 hpf than from 18 hpf. The embryos treated from 12 hpf showed no yolk extension (Figure 2O) or a very short one, whereas the embryos treated from 18 hpf showed only mild shortening of the yolk extension (Figure 2P).

### Functional analysis of *Rbp4 *in zebrafish by morpholino knockdown

**Figure 3:**
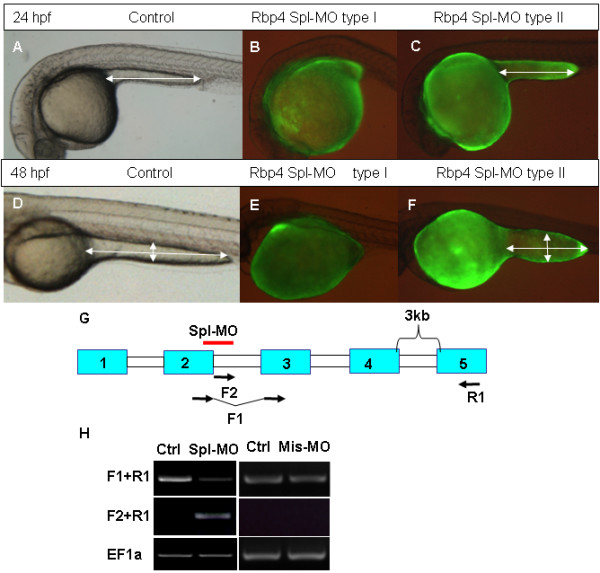
**Effects of Rbp4 splicing MO on morphology of embryos**. Rbp4 Spl-MO was injected into the blastoderm margin area at 4 hpf with Fluorescein and injected embryos were photographed at 24 hpf and 48 hpf. (A, D) Control embryos at 24 hpf and 48 hpf. (B, E) Fluorescent lateral view image of type I phenotype (without yolk cell extension) morphants at 24 hpf (B) and 48 hpf (E). (C, F) fluorescent lateral view image of type II phenotype (short yolk cell extension) morphants at 24 hpf (C) and 48 hpf (F). White double arrows indicate the length and width of yolk extension. (G) Scheme of splicing morpholino and RT-PCR primer positions in the *rbp4 *gene. Exons are represented by blue boxes with numbers and introns by white boxes. A red bar indicates the region targeted by Rbp4 Spl-MO. (H) RT-PCR analysis of RNAs from control (Ctrl) (non-injection)/splcing morphlino injected embryos (Spl-MO) and control (Ctrl) (non-injection)/mismatch morpholino injected embryos (Mis-MO) at 24 hpf using the *rbp4 *primers as indicated in Panel (G) or *EF1a *control primers.

To study the role of *rbp4 *during early development, the morpholino knockdown technique was employed [36,37]. Since the initial expression of *rbp4 *occurs in the YSL, we first focused on a role of this gene in this structure. For this purpose we used the YSL-targeted injection of anti-Rbp4 morpholino (MO) (see Methods for details). Two anti-Rbp4 MOs were designed, one for translation blocking by targeting the 5'-ATG area (ATG-MO) and the other to block splicing by targeting the junction of the 2^nd ^exon and 2^nd ^intron (Spl-MO). After injection of the ATG-MO, the morphants developed relatively normally at least until 16 hpf, but approximately 20% of injected embryos developed a shorter YCE and much larger yolk cell (Table 1). Furthermore, the whole yolk cell of morphants became abnormally dark at 4–5 dpf and they died soon after. Injection of Spl-MO generated morphants with this phenotype more efficiently and in a dose-dependant manner (Table 1). At a low dose (0.4 pmol), the YCE in 58.3% of morphants was shorter than that in controls. At a medium dose (0.8 pmol), 87.3% of morphants displayed abnormal YCE: 77.1% featured short YCE and 10.2% lacked it completely (Figure 3B, C, E and 3F). At a high dose (1.3 pmol), 93.5% of morphants were affected and the fraction lacking YCE increased to 27.3%. To verify the specificity of Spl-MO, a 5 nucleotide mismatched morpholino (Mis-MO) was also synthesized and tested, and there was no noticeable change in the YCE (Table 1).

**Table 1:** Morphological phenotype of *rbp4 *morphants

Phenotype\MO	Total	Type I (no YCE)	Type II (short YCE)	Normal
ATG MO (0.8 pm)	125	0.0%	20.0%	80.0%
Spl MO (0.4 pm)	48	0.0%	58.3%	41.7%
Spl MO (0.8 pm)	166	10.2%	77.1%	12.7%
Spl MO (1.3 pm)	77	27.3%	66.2%	6.5%
Mis-MO(0.8 pm)	65	0.0%	0.0%	100.0%

RT-PCR was used to validate the specificity of Spl-MO. Two sets of primers were designed to monitor the splicing of *rbp4 *transcript: F1 (spanning the junction of exons 2 and 3) + R1 (targeting the 5^th ^exon); F2 (targeting the 2^nd ^intron)+R1 (Figure 3G). When PCR extension time was set at 0.5 minute, F2 +R1 would only amplify the unspliced transcripts and no genomic DNA would be amplified due to presence of the large 4^th ^intron (3 kb). Similarly, primers F1+R1 would amplify only the correctly spliced *rbp4 *transcripts but not genomic DNA or unspliced rbp4 template. As shown in Figure 3H, correctly spliced *rbp4 *transcripts were reduced and the unspliced transcripts were present in 24 hpf morphants (Figure 3H). Sequence analysis also confirmed that the PCR fragment contained the unspliced intron (not shown). Protein sequence prediction from the unspliced transcript showed a premature stop codon after 10 amino acid residues. As a result, the unspliced transcript encoded only the signal peptide. Furthermore, RT-PCR was performed on 24 hpf uninjected control and Mis-MO injected embryos using the same pairs of primers as for the Spl-MO. As shown in Fig 3H, the level of correctly spliced *rbp4 *transcripts in the Mis-MO injected embryos was similar to that of control while unspliced transcripts were not detectable in the Mis-MO injected embryos. Thus, these experimental data demonstrated that the anti-Rbp4 Spl-MO efficiently blocked the processing of *rbp4 *mRNA.

### Liver development in Rbp4 morphants

**Figure 4:**
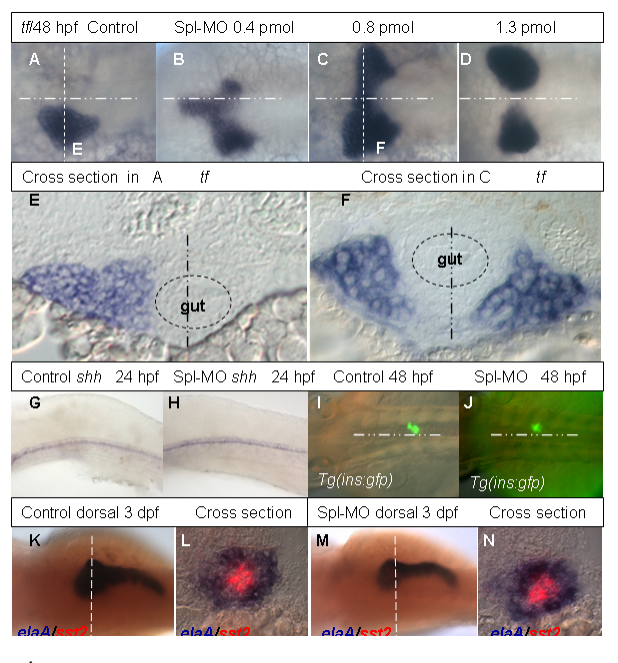
**Analyses of liver and pancreas development in Rbp4 morphants**. (A-D) Dorsal view of *transferrin *expression at 48 hpf in a control embryo (A) and in 48 hpf embryos injected with increasing dosage of Spl MO as indicated (B-D). The midline is indicated by a horizontal point/dash line. (E, F) Cross sections of the control embryo in (A) and morphant in (C) respectively. The section plane is indicated in (A, C) by the vertical dash line. Dashed circles in (E, F) represent gut and the vertical poin/dash lines indicate the midline. (G, H) Lateral view of *shh *expression in 24 hpf control embryo (G) and morphant (H). (I, J) Dorsal view of GFP expression in the principle islet of pancreas in 48 hpf control *Tg(ins:gfp) *embryo (I) and morphant (J). (K-N) Control and morphant stained using two-color WISH with fluorescein-labeled *somatostatin 2*(red) and Dig-labeled *elaA *(blue). Panels (K, M) shows dorsal views of 3 dpf control and morphant respectively. Panels (L, N) are cross section at the planes as indicated in (K, M). Abbreviations: *elaA, elastaseA; sst2, somatostatin 2; shh, sonic hedgehog; tf, transferrin*.

To study the developmental changes in the morphants, several markers expressed in the YSL and endodermal organs were employed. The liver marker *transferrin *[38] indicated two liver buds on both sides of the body axis in embryos injected with either ATG-MO or Spl-MO injection, but not in Mis-MO injected embryos (Table 2). Compared with 27.6% of ATG-MO morphants with two liver buds, a much higher percentage (72.1%) of Spl-MO morphants showed this phenotype (Table 2). The two liver buds were either connected with each other or separated (Figure 4A–D). This phenotype also appeared in a dose-dependant manner; the high dose of Spl-MO caused an increase in a number of morphants with separated liver buds (data not shown). Duplication of liver or other unpaired visceral organs such as pancreas, heart and interrenal gland have been previously observed in mutants with defective midline formation [14,39-41]. In order to examine whether the phenomenon of duplicated liver in Rbp4 morphants was also due to midline defects, several midline markers were evaluated in the morphants at 18 and 24 hpf. *shh *(Figure 4G, H),*no tail *and *twhh *were expressed normally suggesting that formation of the notochord and floor plate was not affected. Therefore the duplication of the liver bud in Rbp4 morphants is not related to defects in midline formation. To further analyze liver morphology, cross-section of 48 hpf morphants was performed. From anterior to posterior, the two liver buds contain several layers of cells similar to that in control (Figure 4E, F). Thus it seems that the two liver buds undergo at least initial stages of hepatogenesis. At the same time, these sections also showed that the gut was normal. In contrast to the YSL-injection, injection of morpholino at 1-cell stage resulted in a lower percentage of the duplicated liver phenotype (5.8%, n = 52). In addition, defects in the brain and tail were observed (data not shown), indicating Rbp4 may have other functions during development. While this study focused on a role of Rbp4 during liver development, other functions of Rbp4 will be analyzed in future.

**Table 2:** Evaluation of *rbp4 *morphants by *transferrin *expression

Phenotype\MO	Total	Two-sided	No-expression	Left-sided
ATGMO (0.8 pm)	58	27.6%	0.0%	72.4%
Spl MO (0.8 pm)	122	72.1%	7.4%	20.5%
Mis-MO (0.8 pm)	16	0.0%	0.0%	100.0%

To analyze development of pancreas, the *Tg(ins:gfp) *that expresses GFP in insulin-producing endocrine pancreatic cells [42] as well as molecular markers for the exocrine (*elastaseA *or *elaA*) and endocrine (*somatostatin2 *or *sst2*) pancreas were used. In both Rbp4 morphants and controls, GFP-positive endocrine β-cells were found on the right side of the midline (Figure 4I, J). Similarly, the *sst2*-positive endocrine cells and *elaA*-positive exocrine cells were found in the same position (Figure 4K–N). Thus, Rbp4 knockdown causes duplication of the liver but not the pancreas. This suggests that Rbp4 probably plays a role only in early liver development. In addition, we never noticed heart duplication in Rbp4 morphants; this observation was further supported by the fact that the expression of *gata6*, an early endoderm and heart marker [43], was not affected at 28 hpf (data not shown).

**Figure 5:**
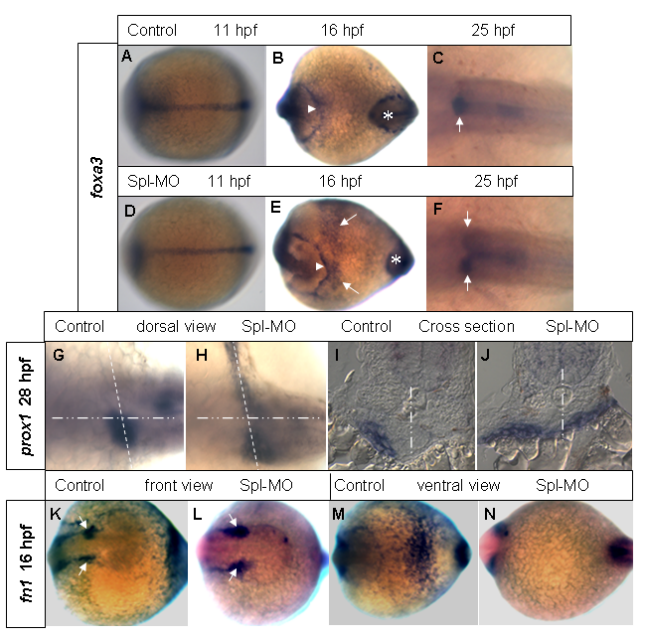
**Rbp4 MO affects the early events of liver patterning prior to formation of liver bud**. (A-F) *foxa3 *expression in both control (A-C) and *rbp4 *morphant (D-F) at 11 hpf (A, D), 16 hpf (B, E) and 25 hpf (C, F) respectively. In (B) and (E), triangles indicate the hatching gland and asterisks the tail bud. The arrows in (E) indicate the ectopic *foxa3 *expression in *rbp4 *morphant. The arrows in (C, F) indicate the liver buds. (G, H) Dorsal view of *prox1 *expression at 28 hpf in whole mount control (G) and morphant (H) embryos. (I, J) Cross section of the embryos as indicated by the dashed lines in (G, H) respectively. The point/dash lines represent midline of the embryos. (K-N), *fn1 *expression at 16 hpf in control (K, M) and *rbp4 *morphant (L, N): front view (K, L) and ventral view (M, N). The arrows in K and L indicate the *fn1 *expression in anterior lateral plate mesoderm.

Migration of endodermal cells towards the midline has been implicated in formation of several visceral organs, including the liver, pancreas, interrenal gland and heart [28,41,14]. Therefore several early endodermal markers were analyzed in morphants. Among these, *foxa3 *is a pan-endodermal marker and is expressed in several endodermal organs including the liver during pharyngula period [44]. As shown in Figure 5D compared to Figure 5A control, there was no altered *foxa3 *expression in Rbp4 morphants at 11 hpf, indicating that the early convergence of anterior endoderm was not affected by knockdown of Rbp4. However, at 16 hpf when there was a major increase of *rbp4 *mRNA in the YSL, in addition to its normal expression in the hatching gland and tail bud [44], *foxa3 *expression in the morphant was observed on the ventro-lateral yolk surface in the morphant (Figure 5E) but not in the control embryos (Figure 5B). Interestingly, this ectopic *foxa3 *expression was detected mainly anteriorly. At later stage, duplicated expression domains in the liver bud regions were observed in the morphant (Figure 5F) but not in the control (Figure 5C). The YSL and lateral plate mesodermal marker *hhex *[45-47] was also used in the study, but there was no obvious alteration of its expression pattern was noticed in Rbp4 morphants (data not shown). Thus, there is no evidence that a defect in endoderm or lateral plate mesoderm caused formation of the two liver buds in the morphants.

To examine the role of *rbp4 *in hepatocyte migration, another early endoderm and hepatocyte marker *prox1 *was used. The homeobox gene *prox1 *is an early marker for the developing liver and pancreas of several vertebrates, including zebrafish, and plays a role in migration of hepatocytes during early liver development of mammals [48,49]. At 28 hpf, a small dot of *prox1 *expression on the left side of the control embryo defined the liver bud, however, in morphants of the same stage, in addition to the *prox1 *expression in the liver bud on the left side, a line-shaped *prox1 *expression domain was found on the right side and it appeared to cross the midline to the left side (Figure 5G, H). Such morphological changes in appearance of endodermal cells have been interpreted as evidence of delayed migration of these cells [14]. In control embryos, the liver bud is located at the A-P level of somites 1–2. The line-shaped *prox1 *staining in morphants was found at the same A-P level. Cross-sections of morphants showed that the *prox1*-positive cells are indeed in the position of the liver bud just above the yolk, but the morphant liver bud is flatter (Figure 5I, J). Based on our current observations, it is likely that early hepatocyte precursor cells migrate into the liver bud and these cells are likely of endodermal origin as they expressed *foxa3 *and *prox1 *endoderm markers. The YSL expression of *rbp4 *appears to be necessary for the migration, which likely occurred from 16 hpf when *rbp4 *expression was largely increased (Fig. 1D) until 25–28 hpf when liver bud formation is completed (Fig. 5G).

Previously it has been reported that the YSL-specific transcription factor Mtx1 regulates myocardial cell migration through downregulation of the extracellular matrix protein Fibronectin1 (Fn1) [14]. To investigate whether the *fn1 *is involved in hepatocyte migration in the Rbp4 pathway, we examined *fn1 *expression in Rbp4 morphants. As shown in Figure 5K–N, *fn1 *was downregulated in the ventro-lateral yolk where *rbp4 *is normally expressed. Interestingly, *fn1 *expression in the myocardial precursor region was not affected in the Rbp4 morphants; consistent with this, no *cardiac bifida *was observed in the morphants. Thus *fn1 *in the ventro-lateral yolk may be specifically involved in yolk extension formation and/or hepatocyte migration.

## Discussion

Rbp4 is a plasma protein acting as a transporter of retinol in blood circulation. During early development *rbp4 *is expressed in the YSL of zebrafish [[18,50], this study]. Because of peculiarities of embryonic development in fish and mammals, direct comparison of the YSL to the extraembryonic structures in mammals is difficult, but comparison of expression of developmental genes may help to solve this puzzle. *Rbp4 *is expressed in the extra-embryonic endoderm of the yolk sac during embryonic development of the rat, mouse and chick [15-17]. Interestingly, zebrafish *rbp4 *is expressed in the YSL on the surface of the yolk cell [[18,50], this study], which, like the yolk sac in mammals, acts as a depot of maternal retinoids in zebrafish [4,5]. These observations suggest functional similarity of these extraembryonic structures in mammals and fish. Further, as suggested by Thomas et al. [51], the YSL plays an important role in early embryonic patterning similar to that of the anterior visceral endoderm (AVE) in mouse embryos [52]. Consistent with this, some murine genes homologous to Xenopus genes important in the organizer (e.g. *hex*, *hesx1, lim1, otx2, cer-1*, etc) are also expressed in the AVE [53]; interestingly, the zebrafish *hhex *is also expressed in the dorsal YSL in zebrafish during gastrula stage [45,54].

Several early developmental genes, including *sqt*, *cas *and *gata5*, are expressed both in the YSL and adjacent vegetal blastomeres [55-58], but the specific roles of these genes in the YSL remain unknown since it is difficult to uncouple these functions from those in the marginal blastoderm. Now it is possible to target the YSL by injecting materials into the YSL only. It has been demonstrated that injections of RNAse into the YSL effectively eliminates YSL transcripts without affecting ubiquitously expressed genes in the blastoderm [13]. By this approach, important information about early function of YSL in the formation of ventro-lateral mesoderm and induction of Nodal-related genes in the ventro-lateral marginal blastomeres has been obtained [13].

However, the specific function of genes expressed in the late YSL remains largely unknown. While a number of genes are expressed in the YSL ubiquitously (e.g. [38,59]), *zangptl2 *is probably the only one with restricted posterior expression pattern in the YSL [29,60]. Our current work on zebrafish *rbp4 *expression in the YSL provides another example illustrating patterning of the YSL. This non-uniform expression pattern suggests that at this stage the YSL could be patterned along the A-P and D-V axes. Consistent with this idea, the distinct morphogenetic domains in the YSL have been reported previously based on migration of its nuclei, though the underlying molecular mechanism remains unknown [61]. The restricted expression of *rbp4 *in the YSL opens a question about mechanisms of such restriction, which we answered to some extent by demonstrating that the Nodal and Hh signalling pathways [62,63] negatively control expression of *rbp4 *similar to that of *ceruloplasmin *[28]. At the same time, RA seems to positively regulate expression of *rbp4 *in the YSL.

The effects of these signalling pathways on liver development require further experimental efforts. In our preliminary experiments, we noticed that both RA and DEAB led to no liver when the treatment was performed before but not after 16 hpf (data not shown). Although RA signalling could regulate *rbp4 *expression, it is difficult to conclude the involvement of Rbp4 in the RA signalling in regulation of liver development because of the pleitropic effect of RA. Nodal and Hh signalling pathways have been reported to play important roles in zebrafish endoderm development [64-71]. In our preliminary analyses using the Nodal and Hh mutants, most of them (*cyc*^-/-^*, smu*^-/-^*, syu*^-/-^*, oep*^-/- ^and *cas*^-/-^) showed either smaller or no liver (data not shown). Only *sqt*^-/- ^showed duplicated liver formation, which is probably due to its midline defect [72] as reported for another midline defect mutant *flh*^-/- ^[40]. However, liver bud duplication in Rbp4 morphant is unlikely due to the midline defect because the midline structure was remained normal in the Rbp4 morphant (Fig. 4G, H).

While it is widely accepted that the YSL plays a leading role during epiboly [73,74], little information is available about the function of YSL after epiboly. It is not known whether the YSL at this stage influences the overlying cells just like that during early gastrulation or, alternatively, the embryo proper influences the patterning of the extraembryonic structures including the YSL. We discovered at least two different functions of Rbp4 within the YSL. First, Rbp4 deficiency results in abnormality of the YCE. Interestingly, *rbp4 *starts to be expressed in the YSL a few hours before the formation of YCE. While the exact molecular mechanism behind the YCE formation is not known, it has been suggested that the YCE formation is influenced by the posterior to anterior migration of cells between the yolk and EVL. These cells accumulate at the level of YCE and could be responsible for YCE formation [29].

Second, Rbp4 is involved in the formation of the liver bud. As Rbp4 is not expressed in the endoderm during this process, its contribution is probably indirect through its role in the YSL. During organogenesis different cell lineages migrate to establish anlage of various organs and differentiate thereafter. Recently it has been reported that the YSL-specific factor Mtx1 plays a role in migration of myocardial precursor cells and posterior endoderm, as knockdown of Mtx1 in the YSL results in *cardia bifida *due to a failure of myocardial cells to migrate to the midline. In parallel, 30% of Mtx1 morphants developed duplicated hepatic and pancreatic buds [14]. Moreover, duplication of liver bud has been also observed in other studies. Ober et al [75] have reported that Vegfc is required for coalescence of anterior endoderm to the midline and knockdown of Vegfc results in formation of a forked gut tube and duplicated buds of liver and pancreas. Similarly, Matsui et al [76] have also reported a new role of non-canonical Wnt signalling during migration of precursor cells toward the midline. The down-regulation of Wnt/Dvl/RhoA signalling leads to the failure of fusion of the anterior gut tube and formation of duplicated livers and pancreas; in addition, migration of myocardial precursors toward the midline is also affected. In contrast to these observations, knockdown of Rbp4 has no effects on migration of heart precursors and the formation of the gut and pancreatic bud. Instead, the deficiency of Rbp4 causes a more limited effect resulting in formation of duplicated liver buds only. Consistent with this, we observed in the Rbp4 morphants that the cell migration molecule Fn1 is specifically reduced in the ventro-lateral region of the yolk, where the *rbp4 *is normally expressed, but not in the myocardia progenitors; Meanwhile, the ectopic expression of *foxa3 *appears specifically in the ventro-lateral region of the yolk. The ventro-lateral increase of *foxa3 *and decrease of *fn1 *suggested that the cells above the *rbp4*-expressing YSL are probable hepatocyte progenitors which will migrate from the ventro-lateral yolk toward the midline depending on a Rbp4-Fn1 signalling pathway. Thus, the effect of Rbp4 is limited probably only to hepatocyte progenitors.

Recently, two conflicting hypotheses of organogenesis of zebrafish visceral organs have been proposed. One emphasizes the formation of the endodermal rod by migration of endodermal cells towards the midline and budding of all major endodermal organs from the rod ([26], reviewed in [25]) while the other puts more weight on the establishment of independent primordia of these organs and their later assembly [27]. Based on the data available, we suggest a unified theory of formation of endodermal organs; i.e. following the formation of the endodermal rod through convergence of the endodermal cells at the midline and the budding of organ primordia, there is continued cell migration from posterior to anterior and from lateral to medial, adding more cells to the buds of organs. Previously, based on analysis of expression pattern of *ceruloplasmin *in the wild type and mutant zebrafish, migration of liver progenitors from both sides of the yolk cell to the midline has been postulated during formation of the liver bud and a role of midline signaling in this process has been illustrated [28]. Migration of liver progenitors is probably a part of a more general process of migration of endodermal cells that contributes also to organogenesis of the pancreas, interrenal gland and heart [14,29,41]. Thus cell migration during the late phase of formation of visceral organs seems to be rather common in zebrafish. Similarly, it has been shown in mice by cell fate mapping that there are different groups of liver precursor cells which migrate to form the liver bud [24]. Our analysis of Rbp4 morphants demonstrated that on the surface of the yolk there are two spatially separated populations of liver precursor cells found on both sides of the midline as proposed earlier [28]. It seems that these cells require an input from the YSL for proper migration. In the present study, we demonstrated that the YSL-expressed Rbp4 is necessary for migration of liver progenitors towards the midline and formation of a single liver bud.

Rbp4 is the extracellular transporter of retinol, a precursor of RA that has been implicated in regulation of cell migration as a stimulator and as an inhibitor of this process depending on the cellular context [77-79]. It stimulates neuronal migration in the zebrafish hindbrain [80]. In view of these earlier observations, our current data suggest that retinoids could play a role in regulating migration of early hepatic cells during the process of liver formation. Given the fact that during the course of our analysis we only evaluated molecular markers and morphology of the heart, pancreas and liver, retinoids could be involved in regulation of cell migration during formation of some other visceral organs that were not analysed here. The developmental roles of Rbp4 in zebrafish revealed in this study should also be considered within a much more general context of metabolism of retinoids in extraembryonic structures that seems to be evolutionarily conserved in all vertebrates studied so far([4,5], reviewed in [81]).

## Conclusion

Rbp4 is traditionally considered to function as a retinol binding protein of the serum. Here we showed that during embryogenesis *rbp4 *mRNA was expressed only in the ventro-lateral YSL and later this expression expanded to cover the posterior YSL in early zebrafish embryos. *rbp4 *expression was negatively regulated by Nodal and Hh signalling and positively controlled by RA. This restricted expression pattern suggested that despite being a syncytium the YSL is patterned. Later *rbp4 *was expressed in the liver. Knockdown of Rbp4 in the YSL resulted in shortened yolk extension as well as the formation of two liver buds, which could be due to impaired migration of liver precursor cells. In contrast, exocrine and endocrine pancreas, intestine and heart developed normally in Rbp4 morphants. Thus we suggest that *rbp4 *expression in the YSL is required only for liver development.

## Methods

### Fish maintenance

Zebrafish were maintained in the fish facilities at the Department of Biological Sciences, National University of Singapore and the Institute of Molecular and Cell Biology in Singapore according to established protocols [82] and in compliance with Institutional Animal Care and Use Committee (IACUC) guidelines. Developmental stages were defined according to Kimmel et al [74] and presented in hours post fertilization (hpf) or days post fertilization (dpf).

### DNA sequencing and analysis

The *rbp4 *cDNA clone (A10) was selected from our zebrafish EST collection [30] and sequenced completely. DNA sequencing reaction was performed using BigDye™ Terminator Cycle Sequencing Ready Reaction Kit (Perkin Elmer, USA) and products of the reaction were separated using the automated sequenator ABI 377 (Perkin Elmer, USA). Sequence analyses and protein identity comparison were performed using DNAMAN^§ ^software.

### RT-PCR

Total RNA was isolated from embryos of selected stages and one-step RT-PCR reaction was conducted using the Qiagen OneStep RT-PCR Kit (Qiagen, Germany). The primers used for amplification of *rbp4 *cDNA were 5'CAGAACGAGGTATCAAGGAA 3' as forward primer and 5'GTCCTCATCCAGCTCTCTGC 3' as reverse primer. The primers for testing the splicing Rbp4 morpholino were as follows: F1, 5'GAGAACGAGGTATCAAGGAAC 3'; F2, 5' GTAAGTCAACCAGTGTTTCC 3'; and R1, 5' CGCGTCTGTATTTGCCCAGG 3'.

### Whole mount in situ hybridization and sectioning

Whole mount *in situ *hybridization using digoxigenin (DIG)-labeled riboprobes was carried out as previously described [83]. The plasmids were linearized with a restriction enzyme followed by *in vitro *transcription with a proper RNA polymerase for antisense RNA probes. Embryos were fixed with 4% paraformaldehyde (PFA), hybridized with the Dig-labeled RNA probe in hybridization buffer (50% formamide, 5XSSC, 50 μg/ml tRNA and 0.1% Tween 20) at 68°C, followed by incubation with anti-Dig antibody conjugated with alkaline phosphatase (AP) and staining with the substrates, NBT (nitro blue tetrazolium) and BCIP (5-bromo, 4-chloro, 3-indolil phosphate).

Two-colour WISH was performed using a combination of the Dig- and fluorescein-labelled probes. For hybridization, two probes were added to the same tube and the embryos were incubated at 68°C overnight. After the first staining with AP-conjugated anti-Fluorescein antibody using Fast Red as substrate to obtain red staining, the embryos were incubated with 0.1 M glycine (pH 2.2) at room temperature for 30 minutes to remove the phosphatase activity. Then the embryos were washed four times (10 minutes each) with PBST (phosphate-buffered saline with 0.1% Tween 20) and incubated in blocking buffer (5% Blocking reagent in malic acid buffer; Roche, Germany) at room temperature for 1 hour. The second staining was performed using AP-conjugated anti-DIG antibody and the substrates, NBT and BCIP, to produce purple precipitates.

For sectioning, the stained embryos were embedded in 1.5% Bactoagar- 5% sucrose and the block containing the embryo was trimmed in desired orientation. The block was then cryoprotected in 30% sucrose at 4°C overnight, mounted into the cryo-mounting media, frozen in liquid nitrogen vapour and sectioned in the cryostat (Microm, Germany). 10–15 μm sections were fixed with 4% PFA in phosphate-buffered saline (PBS) for 10 min, washed with PBS and mounted in 1:1 PBS:glycerol for observation.

### Nuclear Staining

For nuclear staining 10–15 μm sections were equilibrated briefly with phosphate buffered saline (PBS). Approximately 300 μL of 300 nM DAPI (4'6-diamidino-2-phenylindole-dihydrochloride) in PBS was applied to the section and incubated for 5 minutes. The sections were then rinsed several times in PBS and mounted in glycerol/PBS (1:1), followed by observations under a fluorescence microscope (excitation 358 nm, emission 461 nm).

### Morpholino microinjection into YSL

Three Rpb4 antisense norpholino oligonucletides were designed and used in the present study: ATG-MO, 5' GAGCCTTAACATACTGCCTCTGTGC 3'; Sp1-MO, 5' GTTGACTTACCCTCGTTCTGTTAAA 3' and Mis-MO,

5' GTTcACTTAgCgTCcTTCTcTTAAA 3' (the mismatch nucleic acids are in lower case) Microinjection of MO into YSL was performed at 4 hpf stage. The embryos were mounted at proper orientation into pre-made 3% agar wells with egg water (0.3% sea salt, Red Sea brand). The tip of a microinjection pipette was positioned at the blastoderm margin area close to the junction of blastomeres and the yolk. 0.5–1.0 nl of solution containing a defined dose of morpholino and fluorescent marker (Fluorescein or Texas Red labelled 70 kDa dextran) was injected into wild type (AB strain) or *Tg(ins:gfp) *embryos. Usually over 90% of injected embryos showed evenly distributed fluorescent dye in the YSL and these embryos were used for further analyses.

## Authors' contributions

ZL performed all experiments, developed working hypotheses and wrote the manuscript. VK developed a concept of the project and wrote the manuscript. ZG initiated the project and finalized the manuscript. All authors have read and approved the manuscript.
